# Predictive Model for the Diagnosis of Uterine Prolapse Based on Transperineal Ultrasound

**DOI:** 10.3390/tomography8040144

**Published:** 2022-07-01

**Authors:** José Antonio García-Mejido, Zenaida Ramos-Vega, Ana Fernández-Palacín, Carlota Borrero, Maribel Valdivia, Irene Pelayo-Delgado, José Antonio Sainz-Bueno

**Affiliations:** 1 Department of Obstetrics and Gynecology, Valme University Hospital, 41014 Seville, Spain; cbgonzalez@ues.es (C.B.); maribelvaldiviaj@gmail.com (M.V.); jsainz@us.es (J.A.S.-B.); 2 Department of Obstetrics and Gynecology, Faculty of Medicine, University of Seville, 41009 Seville, Spain; 3 Department of Obstetrics and Gynecology, Nuestra Señora de la Merced Hospital, 41640 Seville, Spain; zenaidarv13@hotmail.com; 4 Biostatistics Unit, Department of Preventive Medicine and Public Health, University of Seville, 41009 Seville, Spain; 5 Department of Obstetrics and Gynecology, Ramón y Cajal University Hospital, 28034 Madrid, Spain; ipelayod@yahoo.com; 6 Department of Obstetrics and Gynecology, Faculty of Medicine, University of Alcalá de Henares, 28871 Madrid, Spain

**Keywords:** 3D transperineal ultrasound, pelvic organ prolapse, uterine prolapse, cervical elongation, pelvic floor

## Abstract

We want to describe a model that allows the use of transperineal ultrasound to define the probability of experiencing uterine prolapse (UP). This was a prospective observational study involving 107 patients with UP or cervical elongation (CE) without UP. The ultrasound study was performed using transperineal ultrasound and evaluated the differences in the pubis–uterine fundus distance at rest and with the Valsalva maneuver. We generated different multivariate binary logistic regression models using nonautomated methods to predict UP, including the difference in the pubis–uterine fundus distance at rest and with the Valsalva maneuver. The parameters were added progressively according to their simplicity of use and their predictive capacity for identifying UP. We used two binary logistic regression models to predict UP. Model 1 was based on the difference in the pubis–uterine fundus distance at rest and with the Valsalva maneuver and the age of the patient [AUC: 0.967 (95% CI, 0.939–0.995; *p* < 0.0005)]. Model 2 used the difference in the pubis–uterine fundus distance at rest and with the Valsalva maneuver, age, avulsion and ballooning (AUC: 0.971 (95% CI, 0.945–0.997; *p* < 0.0005)). In conclusion, the model based on the difference in the pubis–uterine fundus distance at rest and with the Valsalva maneuver and the age of the patient could predict 96.7% of patients with UP.

## 1. Introduction

The use of transperineal ultrasound has been used for several aspects of woman pelvis, from surgical follow up of genital prolapse to labor [1,2]. Pelvic floor ultrasound has demonstrated usefulness for the diagnosis of pelvic organ prolapse (POP). A significant ultrasound diagnosis of POP has been defined as the protrusion of the organ of ≥10 mm beyond the posteroinferior margin of the pubic symphysis for the anterior compartment and ≥15 mm for the central and posterior compartment [3,4]. Ultrasound is also useful in the differential diagnosis of the pathology in each compartment, such as different types of cystoceles (Green type I: open retrovesical angle (RVA) ≥ 140°, urethral rotation < 45°; Green type II: open RVA ≥ 140° and urethral rotation 45–120°; Green type III: intact RVA < 140°) in the anterior compartment [5,6,7] or rectocele, enterocele or perineal hypermotility in the posterior compartment [8]. However, the differential diagnosis of the central compartment was ignored until recently, when publications established ultrasound methods for the differential diagnosis of uterine prolapse (UP) and cervical elongation (CE) without UP [9,10].

The diagnosis of POP of the central compartment is based on clinical examination using the International Continence Society Pelvic Organ Prolapse Quantification system (ICS POP-Q) [11]. The prevalence of prolapse, accompanied by loss of vaginal oruterine support, ranges from 6–24% stage 0, to 38–48% stage I, to 35–48% stage II, while 2–6% of the general population have total prolapse beyond the vaginal entrance, stage III [12,13] However, this assessment (ICS POP-Q) has limitations since it only reports on the anatomical surface and uses a mobile soft tissue landmark (the hymen) as a reference point [11]. Therefore, imaging tests have become increasingly important in the diagnosis of POP in this compartment. Magnetic resonance has shown a high agreement for the study of the POP of the central compartment, with comparable findings between clinical evaluation and dynamic magnetic resonance [14]. However, magnetic resonance is not always available to clinicians for the study of POP. In contrast, ultrasound can serve as a fundamental pillar in the diagnosis of POP of the central compartment due to its low cost, easy access, high performance and ability to provide real-time information. Transperineal ultrasound studies have shown that a difference in the pubis–uterine fundus distance at rest and with the Valsalva maneuver of ≥15 mm can diagnose UP with a sensitivity of 75% (95% CI, 64–86%), a specificity of 95% (95% CI, 89–100%), a positive predictive value of 86% (95% CI, 78–95%) and a negative predictive value of 89% (95% CI, 82–97%) [9]. This differential diagnosis of the central compartment is based on a fixed cutoff point (a difference in the pubis–uterine fundus distance at rest and with the Valsalva maneuver ≥ 15 mm) and does not consider other factors that could be associated with UP, such as patient characteristics. Therefore, our objective is to describe a model that, through ultrasound, can define the probability of experiencing UP based on the association between the difference in the pubis–uterine fundus distance at rest and with the Valsalva maneuver associated and the most relevant clinical factors associated with UP.

## 2. Materials and Methods

### 2.1. Subjects

A prospective observational study was conducted at Valme University Hospital of Seville with 107 patients who were recruited consecutively between 1 June 2018, and 31 December 2020. The patients underwent corrective surgery of the middle compartment of the pelvic floor (UP correction or CE without UP). The study was approved by the Biomedical Ethics Committee of the Junta of Andalusia (1259-N-18).

### 2.2. Data Collection

Patients were assessed in consultation through a standardized interview and a clinical examination using the ICS POP-Q system to assess POP [11]. Patients were candidates to undergo corrective surgery of the pelvic floor of the middle compartment were included (Manchester cervical amputation or classic vaginal hysterectomy via the vagina). Patients were assessed in consultation through a standardized interview and a clinical examination using the ICS POP-Q system to assess POP [11]. Prolapse of each compartment was defined as Ba = −0.5, C = −5 and Bp = −0.5 [15]. UP was defined as stage 2 or greater apical compartment prolapse (cases) and CE without UP was defined as C ≥ 0, D ≤ −4, and an estimated cervical length ≥ 5 cm on pelvic examination (controls).

### 2.3. Ultrasound Assessment

**Figure 1:**
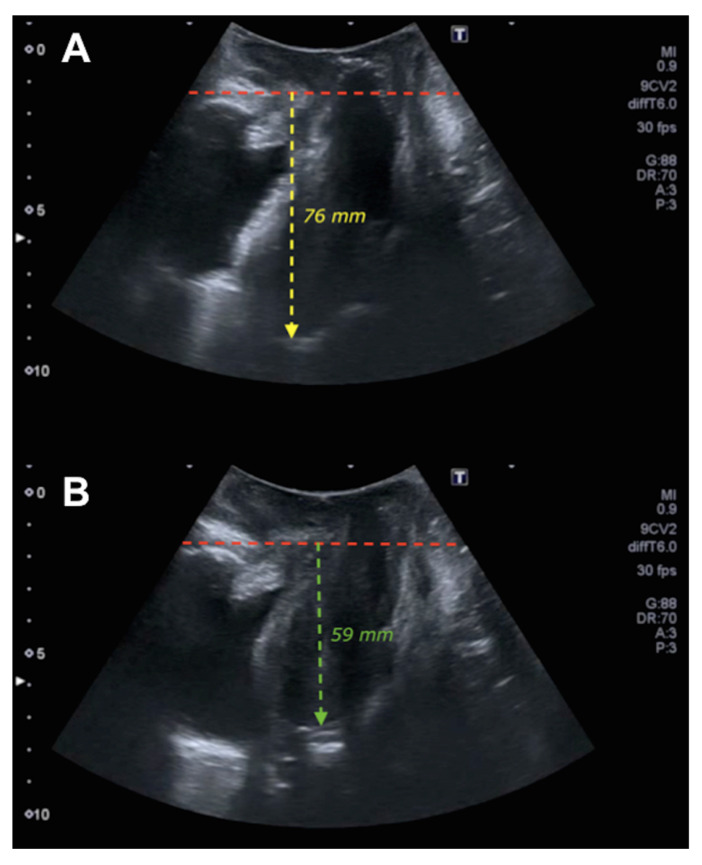
Ultrasound of uterine prolapse. Figure (**A**) shows the midsagittal plane of the pelvic floor at rest where the red line delimits the posteroinferior margin of the pubis and the yellow line the pubis–fundus distance at rest. Figure (**B**) shows the midsagittal plane of the pelvic floor in Valsalva where the red line establishes the posteroinferior margin of the pubis and the green line the pubis–fundus distance in Valsalva.

The ultrasound machines used were a Toshiba^®^ 500 Aplio (Toshiba Medical Systems Corp., Tokyo, Japan) with a PVT-675MV 3-dimensional abdominal probe. Ultrasound examinations were performed according to the previously described methodology [16,17], capturing 3 volumes in each patient 3-4D: at rest, with the Valsalva maneuver (for a minimum of 6 s, assessed with the cine loop preventing the presence of levator coactivation [18]) and with maximum contraction. The degree of POP was assessed using the methodology described in the literature [9] in the midsagittal plane, with the uterine fundus at rest and with the Valsalva maneuver [9] (Figure 1). Measurements within the posteroinferior margin of the pubis were defined as negative values, and measurements outside this margin were defined as positive values [19]. The levator hiatus area (at rest and with Valsalva) was studied in the plane with the smallest hiatal dimension [20]. Levator ani muscle (LAM) avulsion was assessed at maximum contraction using tomographic ultrasound imaging [21,22]. Complete avulsion was diagnosed when abnormal LAM insertion or a levator–urethra gap ≥ 2.5 cm [23] was observed in the three central sections.

### 2.4. Statistical Analysis

We determined means and SDs for numerical variables and percentages for qualitative variables. Comparisons of the numerical variables were performed using Student’s *t*-test. Comparisons of qualitative variables between the study groups were performed using the χ^2^ test. Individual predictive capabilities were evaluated using the receiver operating characteristic curve and the area under the curve (AUC). All statistical comparisons were performed using a two-sided test, and *p* < 0.005 was considered statistically significant for all comparisons. Statistical analyses were performed using the statistical software IBM SPSS version 22 (IBM, Armonk, NY, USA).

#### Evaluation of the Logistic Regression Models

We generated different multivariate binary logistic regression models that used nonautomated methods to predict UP and included the difference in the pubis–uterine fundus distance at rest and with the Valsalva maneuver. The parameters were added progressively to the models according to their simplicity of evaluation and their predictive capacity for identifying UP.

We implemented and compared two binary logistic regression models. We performed a goodness-of-fit test (logarithmic probability of −2) and the Hosmer–Lemeshow test for each model. Then, Harrell’s C statistic (a statistical index used to evaluate the performance of a regression model that analyzes the model’s ability to discriminate between the presence and absence of an event) was determined for the models that had an adequate fit to evaluate their discriminatory capacity (obtained using the AUC of the predicted probabilities given by the model), and the slope and calibration graph were obtained.

The final model was selected based on its ease of application, its discriminatory capacity and its calibration graph, in accordance with the principles of parsimony and interpretability. The models were calibrated by calculating slopes and calibration graphs. Once the definitive multivariate binary logistic regression model was identified, we developed software for predicting UP with the objective of making the model applicable to clinical practice.

## 3. Results

A total of 107 patients were recruited, of whom one patient with UP was excluded due to the difficulty of visualizing the uterine fundus by ultrasound (poor capture of ultrasound volume). A total of 66 patients with UP and 40 patients with CE without UP completed the study. The general and clinical data of the patients who were evaluated and classified according to the presence of UP or CE without UP are shown in Table 1. Statistically significant differences were observed between the two groups in terms of age (62.3 vs. 52.1 years; *p* < 0.0005), the number of deliveries (3.1 vs. 2.1; *p* < 0.0005), the presence of cystocele (77.3% vs. 35.0%; *p* < 0.0005) and the presence of rectocele (24.2 vs. 7.5%; *p* = 0.037).

The ultrasound data according to the presence of UP or CE without UP are shown in Table 2. The levator hiatal area was higher in patients with CE without UP both at rest (20.8 vs. 23.1; *p* = 0.038) and with the Valsalva maneuver (31.2 vs. 33.0; *p* = 0.297). In the patients with UP, LAM avulsion (28.8% vs. 15.0%; *p* = 0.156) occurred more frequently than ballooning (74.2% vs. 87.5%; *p* = 0.139). The pubis–uterine fundus measurement at rest was −66.3 ± 12.8 mm in the UP group and −74.8 ± 16.8 mm in the CE without UP group (*p* = 0.008). The pubis–uterine fundus measurement with the Valsalva maneuver was −41.2 ± 14.8 mm in the UP group and −67.9 ± 17.3 mm in the CE without UP group (*p* < 0.0005). The difference in the pubis–uterine fundus measurement at rest and with the Valsalva maneuver was 25.1 ± 11.7 mm in the UP group and 6.8 ± 4.4 mm in the CE without UP group (*p* < 0.0005).

**Figure 2:**
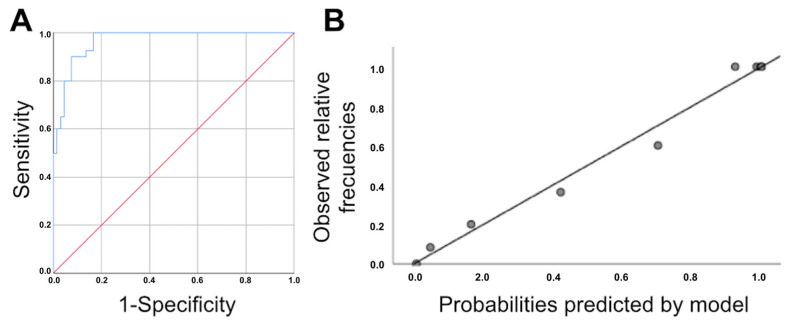
(**A**): ROC curve for the logistic regression model obtained from the association between the difference in the pubis–uterine fundus distance at rest and with the Valsalva maneuver and age. Area under the ROC curve: 0.967 (95% CI, 0.939–0.995; *p* < 0.0005). (**B**): Calibration graph of original logistic regression model obtained for the association between the difference in the pubis–uterine fundus distance at rest and with the Valsalva maneuver and age.

**Figure 3:**
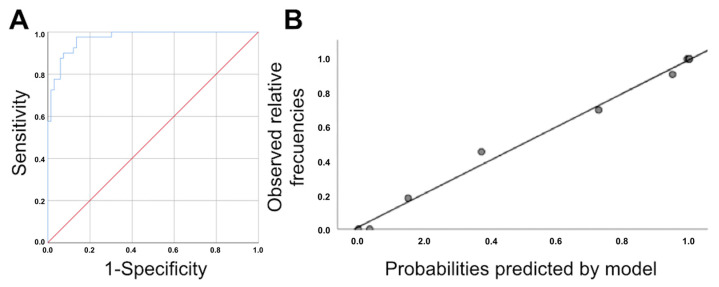
(**A**): ROC curve for the logistic regression model obtained for the association between the difference in the pubis–uterine fundus distance at rest and with the Valsalva maneuver, age, avulsion and ballooning. Area under the ROC curve: 0.971 (95% CI, 0.945–0.997; *p* < 0.0005) (**B**): Calibration graph of the original logistic regression model obtained from the association between the difference in the pubis–uterine fundus distance at rest and with the Valsalva maneuver, age, avulsion and ballooning.

We used two binary logistic regression models to predict UP. Model 1 was based on the difference in the pubis–uterine fundus distance at rest and with the Valsalva maneuver and the age of the patient. Model 2 used the difference in the pubis–uterine fundus distance at rest and with the Valsalva maneuver, age, avulsion and ballooning (Table 3). The Harrell’s C statistic obtained from the AUC of the probabilities predicted by Model 1 was 0.967 (95% CI, 0.939–0.995; *p* < 0.0005) (Figure 2). The calibration of Model 1 was evaluated by calculating calibration slope B, which was 1.004 (95% CI, 0.908–1.101) (Figure 2). The Harrell’s C statistic obtained from the AUC of the probabilities predicted by Model 2 was 0.971 (95% CI, 0.945–0.997; *p* < 0.0005) (Figure 3). The calibration of Model 2 was evaluated by calculating calibration slope B, which was 0.99 (95% CI, 0.925–1.056) (Figure 3). The incorporation of more variables into Model 2 increased predictive capacity with respect to Model 1. However, this increase in predictive capacity was relatively small and required the application of three-dimensional ultrasound parameters (the presence of avulsion and ballooning), which increased the complexity of the examination. Therefore, Model 1 was selected because it had the maximum discriminatory capacity; good calibration, parsimony and interpretability; and was simpler to apply in routine clinical practice.

## 4. Discussion

**Figure 4:**
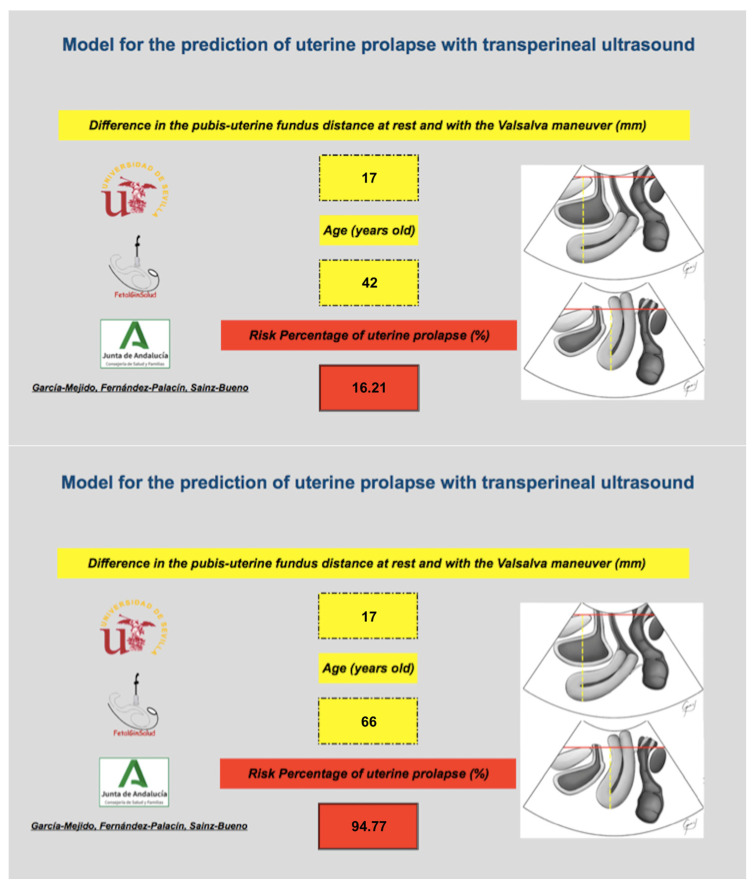
Example of the use of the binary model based on the difference in the pubis–uterine fundus distance at rest and with the Valsalva maneuver and age as a predictor of UP. The image above shows how a 42-year-old patient with a difference in the pubis–uterine fundus distance at rest and with the Valsalva maneuver of 17 mm has a personalized risk of having a PU of 16.2%. The lower image shows how a 66-year-old patient with a difference in the pubis–uterine fundus distance at rest and with the Valsalva maneuver of 17 mm has a personalized risk of having a PU of 94.8%.

The main finding of our study was that Model 1, which was based on the difference in the pubis–uterine fundus distance at rest and with the Valsalva maneuver and the patient’s age, can predict 96.7% of patients with UP. Given the simplicity of this model, which includes only two parameters (the difference in the pubis–uterine fundus distance at rest and with the Valsalva maneuver and age), it is easy to use in the clinic without the need for three-dimensional ultrasound equipment. By applying this predictive model with the available software, any specialist in pelvic floor dysfunctions can easily predict the probability that a patient will experience UP and can optimize the type of surgery for each case (Figure 4). In the example of Figure 1, the difference in the pubis–uterine fundus distance at rest and with the Valsalva maneuver is 17 mm, according to the cut-off point of ≥15 mm [9] the diagnosis of PU would be established. However, when applying the software, this risk varies depending on age (Video S1).

This is the first study to apply software for the diagnosis of UP probability using transperineal ultrasound. Previously, significant prolapse of the central compartment was defined by ultrasound as a protrusion of the cervix more than 15 mm beyond the posteroinferior edge of the pubis during the Valsalva maneuver [4]. However, this methodology does not allow a differential diagnosis of the symptoms of central compartment POP (UP vs. CE without UP). This is because the study that provided this definition did not evaluate the apical fixation points of POP of the central compartment by ultrasound. One of the main differences between UP and CE without UP is that the latter presents a relatively intact DeLancey’s level I (cardinal–uterosacral ligament complex), which is clinically observable with the POP-Q [11]. However, the application of the ICS POP-Q system in patients with central compartment POP has several limitations since it only provides information about the anatomical surface and uses a mobile soft tissue landmark (the hymen) as a reference point. An attempt has been made to assess this reference point with transperineal ultrasound in patients with less than POP-Q stage 2 POP, and clinical assessment was determined to be superior to ultrasound determination [24]. It has also not been reported that 2D transperineal ultrasound is superior to clinical assessment using the POP-Q in the evaluation of symptomatic prolapse [25]. Different associations between the symptoms of central compartment POP and ultrasound findings have been reported, with some studies finding a good correlation (r = 0.77) [26] and others reporting poor results [27]. Despite this, in a recently published multicenter study, it was observed that the concordance of ultrasound with the clinical diagnosis of UP using the ICS POP-Q system was very good at different hospitals, with a kappa index of 0.826 (0.71; 0.94) [28].

Transperineal ultrasound has allowed the differential diagnosis of central compartment POP [28,29]. Related studies have been based on the concept that support of the pelvic organs is related to ligament support associated with the closure of the levator hiatus by the levator ani muscle [30]. Therefore, in cases of apical support failure, a 20% increase in the length of the cardinal ligaments is observed [31]. When we apply these concepts in patients with POP, we observe that the change in the length of these ligaments during the Valsalva in patients with POP is double that of patients with normal support [31]. The identification of patients with apical support outside of the normal range is useful to determine which patients require a hysterectomy and/or an apical support procedure and thereby avoid unnecessary surgical treatments [29]. Consequently, in our work, we have considered the difference between the pubis–uterine fundus distance at rest and with the Valsalva maneuver important for indirectly identifying damaged apical support in UP. Hence, we have established an easy-to-apply model using two-dimensional ultrasound that includes age as a clinical parameter to predict UP.

### Limitations and Strength

The main strength of our study is that it describes a simplified model that evaluates the probability of suffering a UP in a simple, objective manner with high reliability. Additionally, the model assesses the relationship between measurements taken at rest and with the Valsalva maneuver while considering the patient’s age, which allows each patient to be considered individually instead of in relation to a fixed cutoff point, as used in previous studies regarding the diagnosis of significant POP [4]. As a result, with this model, it is possible to adapt the diagnosis to the characteristics of the patient. The study could be criticized for its use of the distance between the uterine fundus and the pubis to assess apical support. We know that apical support is defined by the lower end of the cervix; therefore, we assume that the mobility of the uterine fundus is closely related to the mobility of the lower end of the cervix and thus can be used to assess the fixation of apical support. In addition, there is excellent interobserver reliability for measurements of the difference in the distance from the pubic symphysis to the uterine fundus at rest and during the Valsalva maneuver for both UP and CE without UP [10]. These aspects could support the use of this model in clinical practice. However, external validation is needed before this model can be included in routine consultation. Another limitation to highlight is that the ultrasound study was performed with the patient in the dorsal lithotomy position, and it is possible that this position could limit the protrusion of the POP. However, studies have indicated that there was no difference in the decrease in POP during the Valsalva maneuver when it was assessed in the supine position and in the standing position [32].

## 5. Conclusions

We designed a model based on the difference in the pubis–uterine fundus distance at rest and with the Valsalva maneuver and the age of the patient that can predict 96.7% of patients with UP. We have established a software based on an easy-to-apply model using two-dimensional ultrasound that includes age as a clinical parameter to predict UP.

## Figures and Tables

**Table 1:** General and clinical data of patients assessed and classified according to the presence of uterine prolapse (UP) or cervical elongation (CE) without UP.

	UP (ICS POP-Q) (*n* = 66)	CE without UP (ICS POP-Q) (*n* = 40)	*p*	95% CI
Age	62.3 ± 11.3	52.1 ± 9.9	<0.0005	5.9; 14.4
BMI	27.6 ± 3.3	28.1 ± 4.4	0.464	−2.1; 1.0
Deliveries	3.1 ± 1.6	2.1 ± 0.9	<0.0005	0.4; 1.4
Cesarean sections	0.1 ± 0.5	0.2 ± 0.5	0.384	−0.3; 0.1
Abortions	0.5 ± 0.9	0.7 ± 1.0	0.197	−0.6; 0.1
Age at menopause	52.6 ± 7.9	53.1 ± 5.6	0.790	−5.0; 3.8
Stress incontinence	15 (22.7%)	5 (12.5%)	0.214	−4.5; 25.0
Urge incontinence	22 (33.3%)	8 (20.0%)	0.183	−3.9; 30.6
Mixed incontinence	9 (13.6%)	3 (7.5%)	0.529	−5.8; 18.0
Cystocele	51 (77.3%)	14 (35.0%)	<0.0005	23.9; 60.7
Rectocele	16 (24.2%)	3 (7.5%)	0.037	3.3; 30.2
Enterocele	8 (12.1%)	1 (2.5%)	0.149	−0.2; 20.6

**Table 2:** Ultrasound data according to the presence of uterine prolapse (UP) or cervical elongation (CE) without UP.

	UP (ICS POP-Q) (*n* = 66)	CE without UP (ICS POP-Q) (*n* = 40)	*p*	95% CI
Levator hiatal area (cm^2^)				
Rest	20.8 ± 5.3	23.1 ± 6.1	0.038	−4.6; −0.1
Valsalva	31.2 ± 8.7	33.0 ± 8.5	0.297	−5.2; 1.6
LAM avulsion	19 (28.8%)	6 (15.0%)	0.156	−2.1; 29.7
Ballooning	49 (74.2%)	35 (87.5%)	0.139	−28.3; 1.8
Pubis–uterine fundus measurement				
Rest	−66.3 ± 12.8	−74.8 ± 16.8	0.008	2.3; 14.6
Valsalva	−41.2 ± 14.8	−67.9 ± 17.3	<0.0005	20.5; 33.0
Pubis–uterine fundus measurement. Difference between rest and Valsalva	25.1 ± 11.7	6.8 ± 4.4	<0.0005	15.2; 21.5

**Table 3:** Evaluation of the models.

Models	Variables	OR	95% CI	Calibration (Homer–Lemeshow) *p*	Discrimination (Harrel’s C-Index 95% CI)
1	Pubis–uterine fundus measurement Difference between rest and Valsalva	1.434	1.219–1.688	0.979	0.967 (0.939–0.995)
	Age	1.121	1.041–1.206		
2	Pubis–uterine fundus measurement Difference between rest and Valsalva	1.492	1.243–1.791	0.958	0.971 (0.945–0.997)
	Age	1.124	1.037–1.220		
	LAM avulsion	0.803	0.108–5.944		
	Ballooning	0.120	0.012–1.171		

## Data Availability

The data is kept by the main author.

## Supplementary Materials

The following are available online at https://www.mdpi.com/article/10.3390/tomography8040144/s1, Video S1: title: Practical application of the software.

## Abbreviations

POP	Pelvic organ prolapse
RVA	Retrovesical angle
UP	Uterine prolapse
CE	Cervical elongation
ICS POP-Q	International Continence Society Pelvic Organ Prolapse Quantification system
